# Electroacupuncture inhibits chronification of the acute pain of knee osteoarthritis: study protocol for a randomized controlled trial

**DOI:** 10.1186/s13063-015-0636-y

**Published:** 2015-04-01

**Authors:** Lin-lin Shen, Guo-fu Huang, Wen Tian, Ling-ling Yu, Xiao-cui Yuan, Zhao-qing Zhang, Jing Yin, Chao-yang Ma, Guo-wei Cai, Jian-wu Li, Ming-qiao Ding, Wei He, Xin-yan Gao, Bing Zhu, Xiang-hong Jing, Man Li

**Affiliations:** Department of Neurobiology, School of Basic Medicine, Tongji Medical College of Huazhong University of Science and Technology, No.13 Hang Kong Road, Wuhan, 430030 P. R. China; Institute of Acupuncture and Moxibustion, China Academy of Chinese Medical Sciences, No. 16 Nanxiaojie of Dongzhimennei, Beijing, 100700 P. R. China; Combined Traditional Chinese and Western Medicine Hospital affiliated to Hubei University of Traditional Chinese Medicine, No.215 Zhong Shan Road, Wuhan, 430022 P. R. China; The Third Hospital of Wuhan, No.241 Peng Liuyang Road, Wuhan, 430060 P. R. China; Central Hospital of Wuhan, No.26 Sheng Li Road, Wuhan, 430014 P. R. China; Union Hospital, Tongji Medical College, Huazhong University of Science and Technology, No.1277 Jiefang Ave, Wuhan, 430022 P. R. China; Wuhan Hospital of Traditional Chinese Medicine, No.49 Li Huangpi Road, Wuhan, 430014 P. R. China; The Fifth Hospital of Wuhan, No.122 Xian Zheng Road, Wuhan, 430050 P. R. China

**Keywords:** electroacupuncture, pain, knee osteoarthritis, diffuse noxious inhibitory controls

## Abstract

**Background:**

Previous studies have shown that electroacupuncture (EA) has a significant effect on acute pain, but it has not solved the clinical problem of the chronification of acute pain. Diffuse noxious inhibitory controls (DNIC) function as a reliable indicator to predict the risk of chronic pain events. DNIC function in knee osteoarthritis (KOA) patients has been demonstrated to gradually decrease during the development of chronic pain. The purpose of this study is to conduct a randomized, controlled clinical trial to determine if EA can repair impaired DNIC function and thus prevent chronification of the acute pain of KOA.

**Methods/Design:**

This is a multicenter, single blind, randomized, controlled, three-arm, large-scale clinical trial. A total of 450 KOA patients will be randomly assigned to three groups. The strong EA group will receive EA with high-intensity current (2 mA < current < 5 mA) at the ipsilateral ‘Neixiyan’ (EX-LE5), ‘Dubi’(ST35), ‘Liangqiu’(ST34) and ‘Xuehai’ (SP10). The weak EA group will receive EA with low-intensity current (0 mA < current < 0.5 mA) on the same acupoints. The sham EA group will receive EA with low-intensity current (0 mA < current < 0.5 mA) with fine needles inserted superficially into the sites 2 cm lateral to the above acupoints. The patients will be treated with EA once a day, 30 minutes per session, in 5 sessions per week, for 2 weeks. In order to determine the best stage of KOA for effective EA intervention, patients within the treatment groups also will be divided into four stages. The primary outcomes are Visual Analog Scale (VAS), DNIC function and the Western Ontario and McMaster Universities Osteoarthritis Index (WOMAC). Clinical assessments will be evaluated at baseline (before treatment) and after 5 to 10 sessions of treatment.

**Discussion:**

This trial will be helpful in identifying whether strong EA is more effective than weak EA in reversing chronification of acute pain through repairing the impaired DNIC function and in screening for the best stage of KOA for effective EA intervention.

**Trial registration:**

Chinese Clinical Trial Registry Number: ChiCTR-ICR-14005411. The date of registration is 31 October 2014.

## Background

Knee osteoarthritis (KOA) is one of the most common types of osteoarthritis and often occurs in older adults [1]. Patients with KOA often suffer from pain around the joints and have a high risk of chronification of acute pain. Chronification of acute pain means that persistent acute pain without positive intervention can damage the nervous system and then change into intractable chronic pain [2]. In recent years, a series of clinical randomized controlled trials indicated that electroacupuncture (EA) is effective for treating various painful diseases such as KOA in the acute phase but did not solve the clinical problems of the chronification of acute pain of KOA [3].

The concept of diffuse noxious inhibitory controls (DNIC) has attracted particular attention in the past decades, which means under normal conditions, pain can be attenuated by a conditioning noxious stimulus to a remote body region [4]. DNIC function is now regarded as a reliable indicator for predicting the risk of chronic pain [5,6]. DNIC function in KOA patients has been shown to gradually decrease with the development of chronic pain [7]. Thus, we hypothesized that the process of chronification of acute pain may be related to gradually impaired DNIC function, and EA may prevent the occurrence of chronification of acute pain by improving DNIC function.

The aim of this study is to explore whether EA can reverse impaired DNIC function by reducing the pain of KOA during the process of chronification of acute pain. Moreover, we will screen for the best EA therapeutic regimen, which will include optimizing the intensity of the current used in the EA stimulation and determining the best stage for effective EA intervention using a randomized controlled clinical trial.

## Methods/Design

### Ethics

The study is in accordance with the Declaration of Helsinki, it has been approved by the Chinese Ethics Committee of Registering Clinical Trials (reference: ChiECRCT-20140035), and it also has been registered with Chinese Clinical Trial Registry (ChiCTR-ICR-14005411). Before randomization, all patients will be expected to sign a written informed consent.

### Design

**Figure 1:**
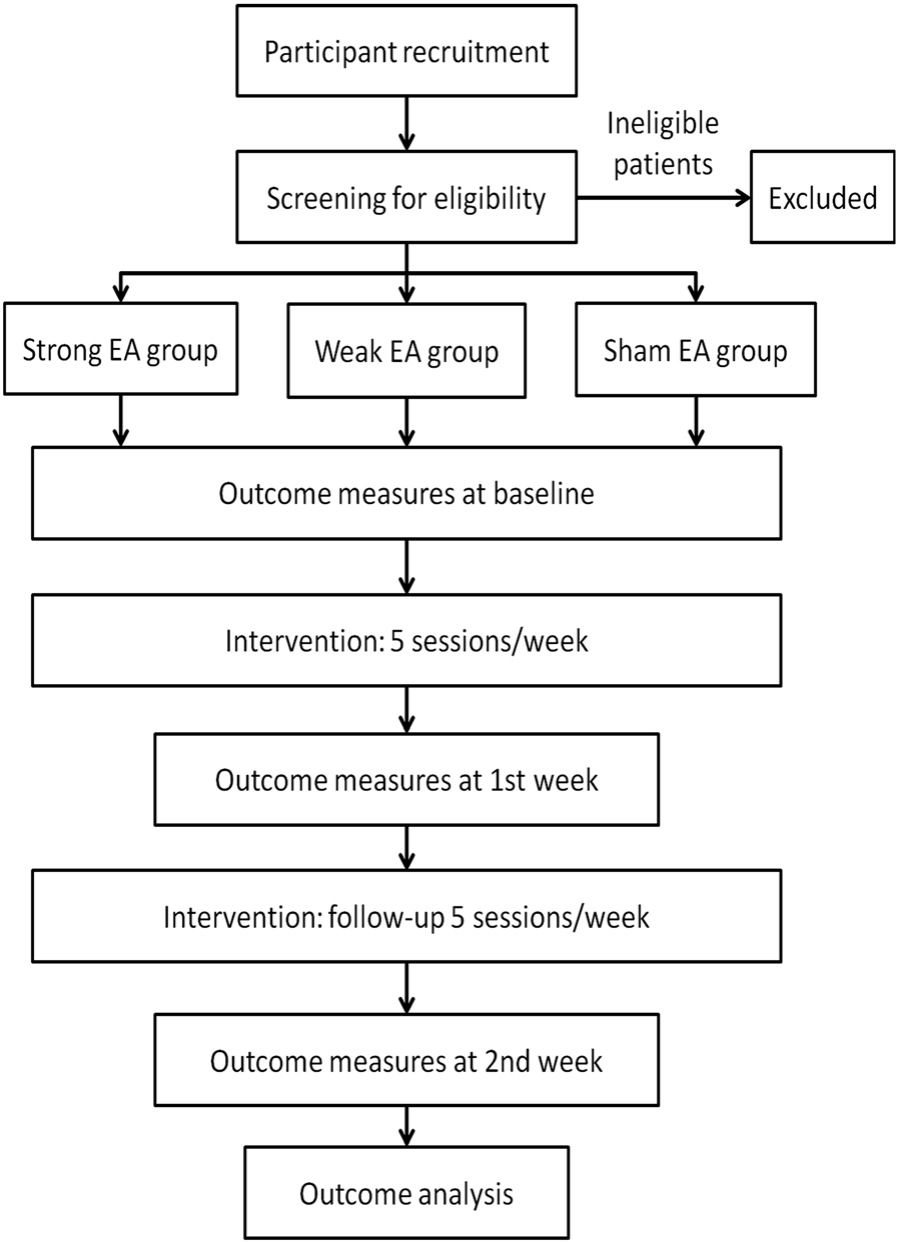
**Trial flow chart.**

A randomized, controlled, three-arm, large-scale trial to compare two true EA groups (strong EA and weak EA) with a sham EA group will be undertaken in this study [8]. Four hundred and fifty KOA patients will be recruited from the following six hospitals: the Combined Traditional Chinese and Western Medicine Hospital Affiliated to Huazhong University of Science and Technology, The Third Hospital of Wuhan, Central Hospital of Wuhan, Union Hospital Affiliated to Tongji Medical College of Huazhong University of Science and Technology, Wuhan Hospital of Traditional Chinese Medicine, and The Fifth Hospital of Wuhan. Patients will be randomized in a ratio of 1:1:1 to the strong EA group, weak EA group and sham EA group. After submitting informed consent and being randomized, patients will receive 10 sessions of EA treatment over a period of 2 weeks, with a treatment frequency of 5 sessions per week. Each session will last 30 minutes. Patients will be asked to accept assessments at baseline, as well as at the end of the first and second weeks of the treatment phase. Time points and groups are shown in Figure 1.

### Randomization and blinding

Patients who meet the inclusion criteria will be randomly assigned to one of three groups (strong EA, weak EA or sham EA) in a ratio of 1:1:1 using a computer-generated random allocation sequence through the stratified block randomization method of SAS version 9.1.3 (SAS Institute Inc., USA). The randomization will be performed by an independent research assistant, who will inform the acupuncturists of the treatment assignments by mobile message. Allocation concealment will not be exposed until the final data analysis report is completed.

To maintain blinding, each patient will have needles (true or sham) inserted into his/her ipsilateral leg. The needles are connected to an EA apparatus, and the patients can feel electric stimulation. Strong and weak EA groups will be acupunctured on four acupoints. The sham EA group will be acupunctured on four non-acupoints without ‘De-qi’.

In this clinical trial process, the patients, statisticians and the evaluators for the statistical data and outcomes are blind to the treatment allocation. However, the blind method is not used by acupuncturists in the implementation of EA manipulation because it is not feasible to conceal allocation from them.

### Patients

#### Study population

Patients will be recruited from among acupuncture inpatients and clinics of six hospitals with a target sample size of 450 subjects. The trial will be ongoing from September 2014 to September 2016.

#### Inclusion criteria

Patients who meet the clinical criteria for KOA formulated by the American College of Rheumatology (ACR) will be considered for inclusion [9]. According to the ACR, clinical KOA is defined as knee pain and at least three out of seven of the following criteria: patient is older than 50 years of age, morning stiffness lasts less than 30 minutes, crepitus is present, bony enlargement is apparent, bony tenderness is present, no palpable warmth is present, and presentation is suggestive of a radiological osteophyte.

#### Exclusion criteria

The exclusion criteria are as follows:

1. Patient has had an adverse reaction to acupuncture or is unwilling to accept acupuncture treatment.
2. Patient conforms to the inclusion criteria, but does not follow prescribed treatment, which decreases the curative effects of EA so that it cannot be judged, or patient has incomplete information that may interfere with his/her ability to accurately judge the effects of his/her treatment.
3. Patient has accompanying severe cardiovascular, cerebral, hepatic, renal, or hemopoietic diseases [10].
4. Patient has inflammatory arthritis such as rheumatoid arthritis, gouty arthritis, *etcetera* or other diseases that may affect the condition of the knees [11].
5. Patient is pregnant, attempting to become pregnant or lactating [10].
6. Patient has a mental disease [11].

#### Recruitment of patients

Two schemes will be used to recruit patients with KOA. The first is to recruit patients in the outpatient and inpatient departments from the six hospitals. The chief physicians of the acupuncture and rehabilitation department in each hospital will screen eligible patients according to the inclusion/exclusion criteria with the help of research assistants. Moreover, potential patients in local communities and out-hospital clinics will be recruited by advertisements through posters, leaflets, newspapers, and so on. After being screened and found to meet the inclusion criteria, the patients will be included only if they agree to join in every procedure of the trial willingly and provide written informed consent.

### Interventions

#### Electroacupuncture group

**Figure 2:**
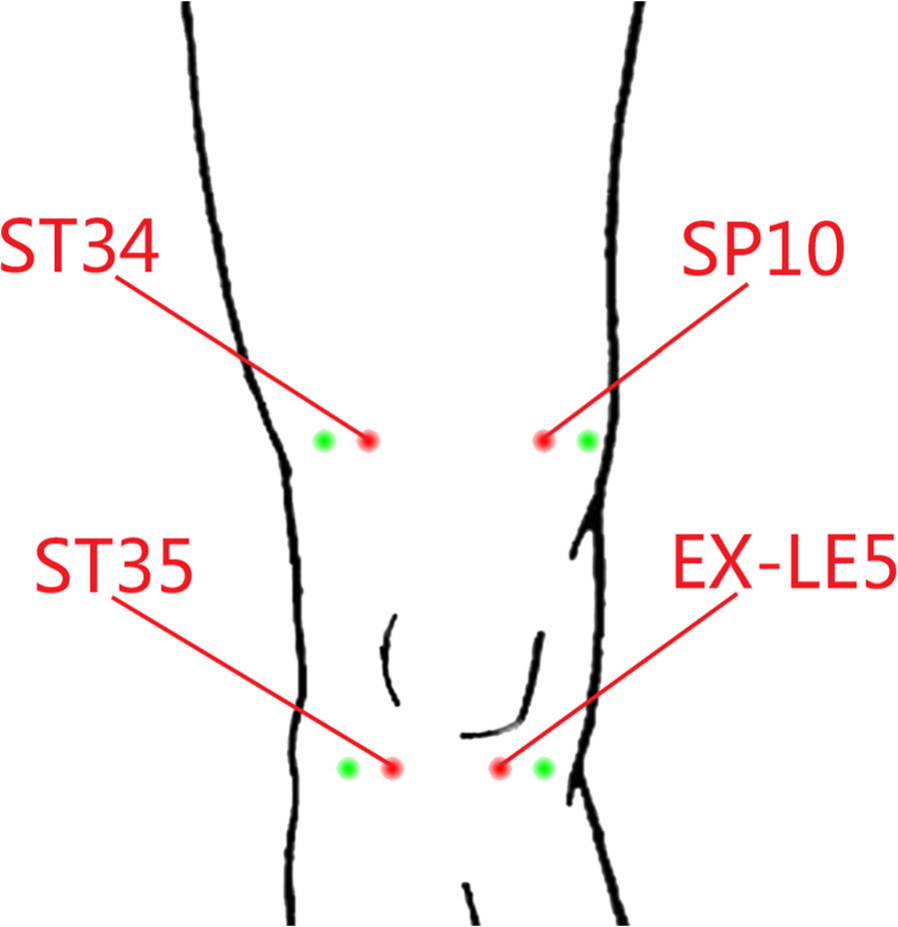
**The points used in the trial.** The lines refer to acupoints of ‘Liangqiu’ (ST34) and ‘Dubi’ (ST35) on the lateral side of the knee, and ‘Xuehai’ (SP10) and ‘Neixiyan’ (EX-LE5) on the medial side of the knee. The points lateral to the above four acupoints refer to non-acupoints.

According to the Traditional Chinese Medicine meridian theory of treating pain of KOA [11,12], patients in the strong and weak EA treatment groups will be treated with EA on ipsilateral acupoints of ‘Neixiyan’ (EX-LE5), ‘Dubi’(ST35), ‘Liangqiu’(ST34) and ‘Xuehai’ (SP10) once a day (Figure 2). The sterile, disposable Hwato needles used in this trial are made in Suzhou, China. Patients are positioned on the bed, supported by two pillows under the knees and instructed to assume a comfortable position and not to move during the 30-minutes stimulation period.

After the local area has been disinfected, the needles (30-gauge with an outer diameter of 0.32 mm and a length of 50 mm) will be inserted at a depth of 25 to 40 mm vertically and ‘De-qi’ (the acupuncture sensation of fullness, heaviness, dull aching or warmth) sensation will be achieved in the strong and weak EA groups through lifting and thrusting combined with twirling and rotating the needles. Direct current and continuous wave will be delivered with a medical EA apparatus (model G9805, Shanghai, China), at 2 Hz frequency and 0.5 ms pulse width for 30 minutes. EX-LE5 will be connected to ST35, and SP10 will be connected to ST34 with a pair of electrodes [10,13]. Different stimulation intensities will be allocated to the strong EA and weak EA groups. The strong EA group will receive the maximum tolerable intensity of current (2 mA < current < 5 mA). However, patients in the weak EA group will receive low-intensity current (0 mA < current < 0.5 mA), and they will be instructed to ask the doctor to stop the increase in intensity when the sensations of current occur. After the intervention period, all needles and the EA apparatus will be removed (Table 1).

**Table 1:** **Details of each group**

**Group**	**Acupoints**	**Stimulation parameters**
Strong EA group	Neixiyan	Direct current, continuous wave, at 2 Hz frequency and with 0.5 ms pulse width for 30 minutes. Maximum tolerable intensity of current will be delivered, 2 mA < current < 5 mA. EX-LE5 will be connected to ST35 and SP10 will be connected to ST34 with a pair of electrodes.
	(EX-LE5)	
	Dubi (ST35)	
	Liangqiu (ST34)	
	Xuehai (SP10)	
Weak EA group	Neixiyan	Stimulation indexes and acupoint’ connections are the same as in the strong EA group, but the patients will just feel the sensations of current, 0 mA < current <0.5 mA.
	(EX-LE5)	
	Dubi (ST35)	
	Liangqiu (ST34)	
	Xuehai (SP10)	
Sham EA group	The sites are 2 cm lateral to the above	Fine needles will be inserted superficially into the sites 2 cm lateral to the above four points with low-intensity current as same as in the weak EA group.
	four points	

Note: EA, electroacupuncture.

#### Sham EA group

In the sham EA group, a sterile, disposable, fine and short needle (35-gauge needle with an outer diameter of 0.20 mm and a length of 25 mm) will be used and inserted superficially (to an approximate depth of 5 to 10 mm) into the sites of the non-acupoints 2 cm lateral to the above four acupoints (Figure 2). However, the ‘De-qi’ sensation will not be intentionally elicited in this group. Electrical stimulation will be delivered with low-intensity current (0 mA < current < 0.5 mA) at 2 Hz frequency and 0.5 ms pulse width for 30 minutes, which is the same as for the weak EA group (Table 1).

#### Education of practitioners

All EA and sham EA treatments will be performed by acupuncturists who have been qualified for at least 3 years and hold Chinese medicine practitioner licenses from the Ministry of Health of the Peoples Republic of China. Each acupuncturist will take a pretrial training course for this clinical study. The chief physicians will train all acupuncturists and observe their techniques periodically to ensure standardization. In addition, all study protocols and details, including the recording method for the case report form, outcome assessment methods, and monitoring process, will be standardized among the six centers by the research assistants.

### Outcome assessments

#### Primary outcome measurements

The primary outcome measurements are the Visual Analog Scale (VAS), DNIC function and the Western Ontario and McMaster Universities Osteoarthritis Index (WOMAC).

VAS is an internationally recognized pain scale. It is a 100-mm line ranging from 0 (no pain) to 100 (pain as bad as it could be) [14].

**Figure 3:**
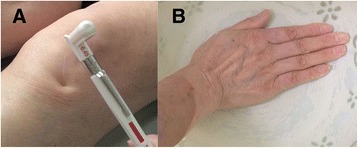
**The main procedure for the measurement of diffuse noxious inhibitory controls (DNIC) function. A.** On the Ashi-point of the knee, a 180-g von Frey filament is used to prick three to five times within a 1-cm-diameter circle at an angle of 45-degrees between the filament and the skin. The patients will be asked to rate the intensity of pain as VAS1. **B.**The patients will be asked to immerse the contralateral hand, including the wrist, into nociceptive cold water (10 to 12°C) for 1 minute, and the doctors will rate the intensity of the 180-g von Frey filament-induced pain at the Ashi-point as VAS2. DNIC = (VAS1-VAS2)/VAS1.

The prediction of chronification of acute pain can be indicated by DNIC function, which means that pain elicited by a noxious stimulus applied to a given body site can be attenuated by application of a conditioning noxious stimulation to another, even distant body region [15]. DNIC function will be measured according to the procedure shown in Figure 3. On the Ashi-point (pain spot) of the affected knee, the research assistant will apply a 180-g von Frey filament (bending it so that it forms a 45-degrees angle with the skin) three to five times within a 1-cm-diameter circle (Figure 3A). The patients will be asked to rate the intensity of pain as VAS1 [5], which is the induced mechanical pain score of the affected knee. Then, the patients will be asked to immerse the contralateral hand, including the wrist, into nociceptive cold water (10 to 12°C) for 1 minute and rate the intensity of the pain induced by the 180-g von Frey filament on the Ashi-point as VAS2 (Figure 3B). For healthy people, the conditioning cold noxious stimulation can activate the descending inhibitory system of the spinal cord. VAS2 is used to detect the induced mechanical pain score of the affected knee after conditioning with the cold noxious stimulation on the contralateral hand. Finally, DNIC function will be assessed by measuring the difference in pain ratings before and after immersion (DNIC = (VAS1-VAS2)/VAS1). Thus, the decreased ratio in pain scores of the affected knee before and after the cold noxious stimulation of the hand is the DNIC function [2,16].

WOMAC can be used as an initial assessment of the intensity of pain and functional disability of KOA [3]. It is a multidimensional measure of pain, stiffness, and physical functional disability [17]. Briefly, five questions will be asked about pain dimension at activity or rest. The stiffness dimension includes two questions. Questions about the degree of difficulty in 17 different activities will be asked for the physical functional disability dimension. All 24 questions will be listed in a numerical rating scale ranging from 0 (no symptoms) to 3 (maximum symptoms). Doctors need to help patients perform the self-assessment, collect the total score from the 24 questions and record it.

#### Secondary outcome measurements

The secondary outcome measurements are Numerical Rating Scale (NRS), Emotional Scale (ES), Present Pain Intensity (PPI) and range of motion (ROM).

The NRS of pain is a scale containing the numbers from 1 to 10 on a straight line to measure the magnitude or intensity of pain [18]. It has been shown to have a good predictive validity and has been used to measure the effect of EA on the pain of KOA [12]. All subjects participating in the study will be asked to select a number from 1 to 10 with the nearest 0.5 interval, to represent their maximal OA-induced knee pain in the most recent 2 to 3 days.

Pain, whether acute or chronic, can affect a patient’s emotions. Under normal circumstances, emotion can be distinguished according to four levels, measured as ES: Excellent (0 to 2, face is peaceful, and the patient responds freely), Good (3 to 5, face remains indifferent, and the patient responds to instruction), General (5 to 8, anxiety or depression apparent, face shows some pain, and patient barely answers), and Poor (8 to 10, face clearly shows pain, with moaning and forced posture, and patient fails to answer).

PPI is also one of the clinical tools most commonly used to help patients express the intensity of pain. It moves from 0 to 5, indicating the following levels of pain according to number: no pain, mild discomfort, discomfort, terrible pain and extreme pain.

ROM indicates the maximum range of joint activities. Determination of ROM is a basic step in assessing muscle, bone and neurological damage. It is one of the indicators used to evaluate the scope of damage and degree of joint movement function, and it can be measured with the help of protractor whose axis is placed on the fibular capitulum, with the fixed arm parallel to the long axis of the femur and with the moving arm parallel to the long axis of the fibula while in a prone position. The normal range of motion of the knee is ‘0 to 135°’.

### Sample size

According to a previous study and our pilot data, the sample size calculation assumed an average DNIC increase percentage of 30% for the strong EA group [19]. The DNIC increase percentage for the weak EA group and sham EA group are assumed to be 20% and 15%, respectively. It is assumed that 15% of the trial patients will be lost to attrition. With this scenario, 150 patients need to be allocated to each treatment group to establish a difference among the treatments at a 5% level and with a power of 90% [20].

### Data analysis

Data with normal distribution will be presented as means ± SEM. We will use a one-way analysis of variance (ANOVA) or a two-way ANOVA to determine the overall effect of EA. The Post hoc test (Newman Keuls test) will then be used to determine the statistical differences among individual groups. A *P* value of less than 0.05 will be considered statistically significant. Data showing an abnormal distribution will be analyzed by the Wilcoxon signed-rank test.

**Figure 4:**
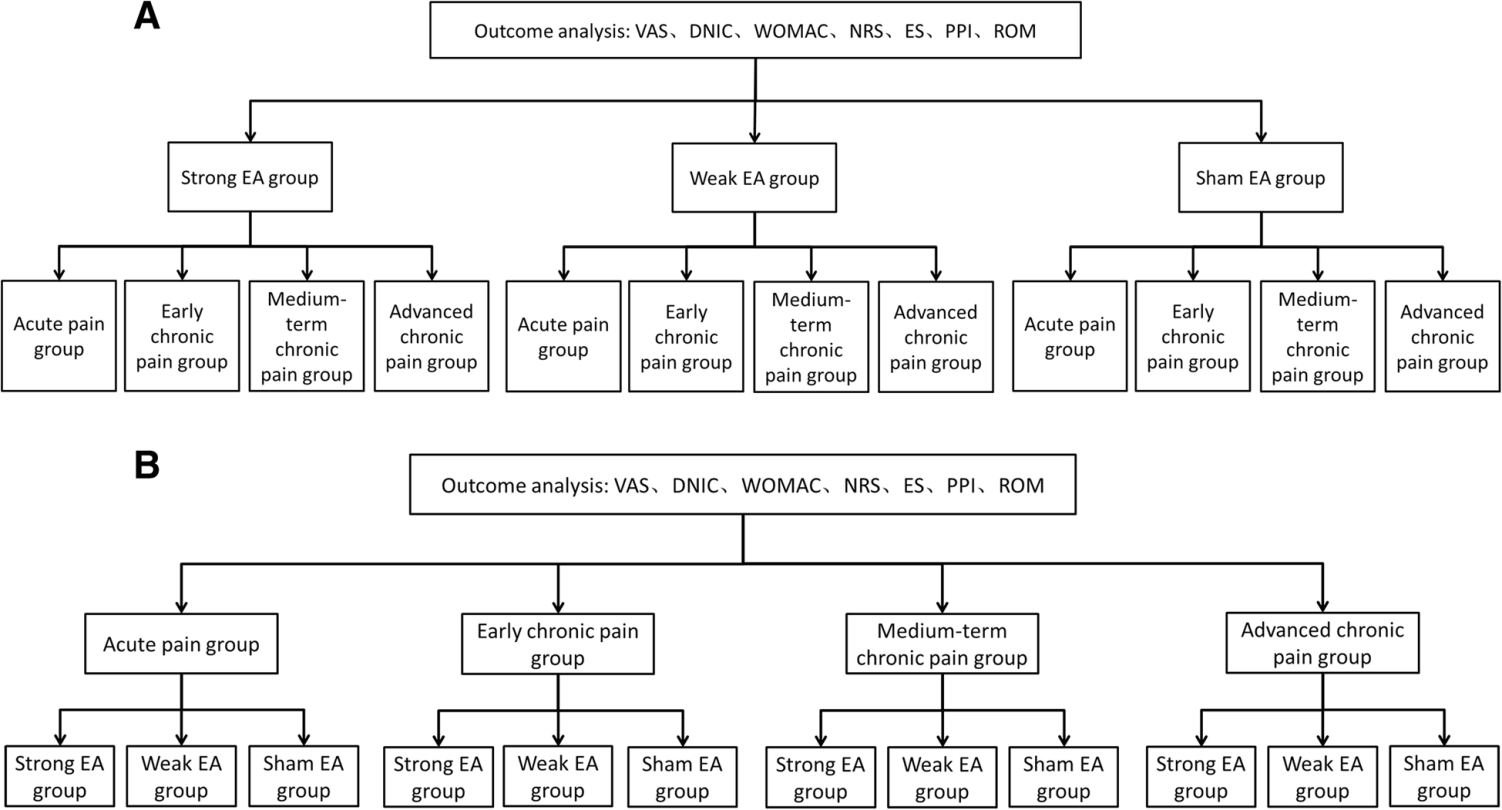
**Data analysis chart. A**. We will divide the three electroacupuncture (EA) groups into four different stages to screen separately for the stage where the EA intervention will be most effective. **B**. We will divide all patients into four stages and compare the effects of strong, weak and sham EA separately within the different stages. The Visual Analog Scale (VAS), diffuse noxious inhibitory control (DNIC) function, Western Ontario and McMaster Universities Osteoarthritis index (WOMAC), Numerical Rating Scale (NRS), Emotional Scale (ES), Present Pain Intensity (PPI), range of motion (ROM) will be measured by the procedure outlined in the chart.

In order to determine which EA intervention is the best for each of the different stages of KOA, patients in the three EA groups will be further divided according to four different stages of KOA:

1. The acute pain stage group. The pain of KOA has been ongoing for less than 6 months.
2. The early chronic pain stage group. The pain of KOA has been ongoing for 6 months to 3 years.
3. The medium-term chronic pain stage group. The pain of KOA has been ongoing for 3 to 5 years.
4. The advanced chronic pain stage group. The pain of KOA has been ongoing for more than 5 years (Figure 4A).

The effect of EA on the different stages of KOA will be observed, and the stage with the best response to EA intervention will be discussed. Furthermore, in yet another comparison, we will divide all patients into the four stages and then compare the effects of the strong, weak and sham EA groups within the different stages separately (Figure 4B).

## Discussion

The aim of this trial is to use EA intervention to inhibit chronification of the acute pain of KOA by reversing the damage to the DNIC function. Completion of this trial will help to identify whether EA with high-intensity current (strong EA) is more effective than EA with low-intensity current (weak EA) and whether EA applied at an early stage of KOA will be more effective than that at a later stage of KOA.

We will establish two treatment groups and one control group in this randomized controlled trial. Since the primary consideration of this trial is to clarify whether EA is effective in preventing chronification of the acute pain of KOA, we will use sham EA as a control to rule out the placebo effect. In the sham EA group, acupuncture needles are shallowly inserted into non-acupoint locations to rule out the effect of acupoint. Because most of the KOA patients are familiar with the sensation of EA, they could realize that it is the sham EA if they cannot feel any current from the apparatus. Therefore, we will use a low intensity of electrostimulation to exclude a placebo effect.

Acupoints of EX-LE5, ST34, ST35, and SP10 are located around the knees, which have been regarded as the most common and effective acupoints for use in treating KOA in many high-quality trials [11,21]. Since 2 Hz is the optimized EA frequency for the relief of inflammatory pain, we chose the 2 Hz as the best frequency of EA to treat KOA in this study [22].

DNIC involves supraspinal structures, modulates the transmission of nociceptive signals and is another pain perception methodology recently used to study pathophysiology of various pain disorders because it reflects the function of the endogenous pain system [23,24]. This pain-modulating process is the neurophysiological basis for the well-known phenomenon where ‘pain inhibits pain’ from remote areas of the body [5]. The continuous pain input will impair the DNIC function and then decrease antinociceptive activity from the supraspinal structures, which may result in the occurrence of chronic pain [25]. Numerous studies have demonstrated that DNIC may be involved in the analgesic mechanism of acupuncture [26,27]. However, no study has used DNIC function to screen for the best EA intensity and for the best stage of chronification of acute pain for an effective EA intervention.

To screen for the optimal intensity of EA for improving DNIC function, the effect of strong EA and weak EA will be compared. EA with low-intensity current (less than 1 mA) has been demonstrated to mainly excite large fibers (Aβ fiber), which may not activate the DNIC function [28]. Instead, this innocuous stimulation may only exert an analgesic effect through the spinal mechanism of gate control theory [29]. In contrast, EA with high-intensity current (more than 2 mA) excited thin fibers (A δ and/or C fibers) and may activate DNIC function [30]. Thus, we will compare the effect of strong EA and weak EA on repairing impaired DNIC function to screen for the best EA intensity to prevent chronification of the acute pain of KOA. We hypothesize that both strong EA and weak EA may have some pain-reducing and inflammation-relieving effects, which will be assessed with VAS, WOMAC, NRS, ES, PPI and ROM. However, strong EA may be more effective in improving DNIC function and, thus, in reversing the chronification of acute pain [28].

Moreover, we also will try to determine the best stage of KOA for EA intervention. DNIC function is the function of the endogenous analgesic system [16]. In other words, the process of gradually decreased DNIC function is also the process of transforming acute pain to chronic pain. In this process, if patients receive EA intervention as soon as possible, it is possible that the chronification of pain in KOA patients will be prevented. However, at the later stages of chronic pain, peripheral and central sensitization will likely irreversibly damage the endogenous analgesic system function and completely impair DNIC function [2]. At that point, EA may not be as effective. So, we will further divide the KOA patients according to the stage of the disease. We hope to find the best stage for the use of EA to reverse the impaired DNIC function and prevent the chronification of acute pain.

This project will not only increase knowledge on the effects of treating KOA with EA, but also may contribute a solution to the difficult problem of the chronification of acute pain, and promote the clinical application of EA analgesia.

## Trial status

This trial is currently recruiting patients.

## Abbreviations

ACR: American College of Rheumatology
DNIC: diffuse noxious inhibitory controls
EA: electroacupuncture
ES: Emotional Scale
KOA: knee osteoarthritis
NRS: Numerical Rating scale
PPI: Present Pain Intensity
ROM: range of motion
VAS: Visual Analog Scale
WOMAC: Western Ontario and McMaster Universities Osteoarthritis Index
